# Saturated fatty acids differently affect mitochondrial function and the intestinal epithelial barrier depending on their chain length in the *in vitro* model of IPEC-J2 enterocytes

**DOI:** 10.3389/fcell.2024.1266842

**Published:** 2024-02-01

**Authors:** Thomas Guerbette, Vincent Rioux, Mégane Bostoën, Vincent Ciesielski, Hugo Coppens-Exandier, Marine Buraud, Annaïg Lan, Gaëlle Boudry

**Affiliations:** ^1^ Institut NuMeCan, INRAE, INSERM, University Rennes, Rennes, France; ^2^ Institut Agro Rennes-Angers, Rennes, France; ^3^ UMR PNCA, AgroParisTech, INRAE, Université Paris-Saclay, Paris, France

**Keywords:** intestine, mitochondria, barrier function, metabolism, fatty acids, enterocyte

## Abstract

**Introduction:** Maintenance of the intestinal barrier mainly relies on the mitochondrial function of intestinal epithelial cells that provide ATP through oxidative phosphorylation (OXPHOS). Dietary fatty acid overload might induce mitochondrial dysfunction of enterocytes and may increase intestinal permeability as indicated by previous *in vitro* studies with palmitic acid (C16:0). Yet the impact of other dietary saturated fatty acids remains poorly described.

**Methods:** To address this question, the *in vitro* model of porcine enterocytes IPEC-J2 was treated for 3 days with 250 µM of lauric (C12:0), myristic (C14:0), palmitic (C16:0) or stearic (C18:0) acids.

**Results and discussion:** Measurement of the transepithelial electrical resistance, reflecting tight junction integrity, revealed that only C16:0 and C18:0 increased epithelial permeability, without modifying the expression of genes encoding tight junction proteins. Bioenergetic measurements indicated that C16:0 and C18:0 were barely β-oxidized by IPEC-J2. However, they rather induced significant OXPHOS uncoupling and reduced ATP production compared to C12:0 and C14:0. These bioenergetic alterations were associated with elevated mitochondrial reactive oxygen species production and mitochondrial fission. Although C12:0 and C14:0 treatment induced significant lipid storage and enhanced fusion of the mitochondrial network, it only mildly decreased ATP production without altering epithelial barrier. These results point out that the longer chain fatty acids C16:0 and C18:0 increased intestinal permeability, contrary to C12:0 and C14:0. In addition, C16:0 and C18:0 induced an important energy deprivation, notably via increased proton leaks, mitochondrial remodeling, and elevated ROS production in enterocytes compared to C12:0 and C14:0.

## Graphical Abstract

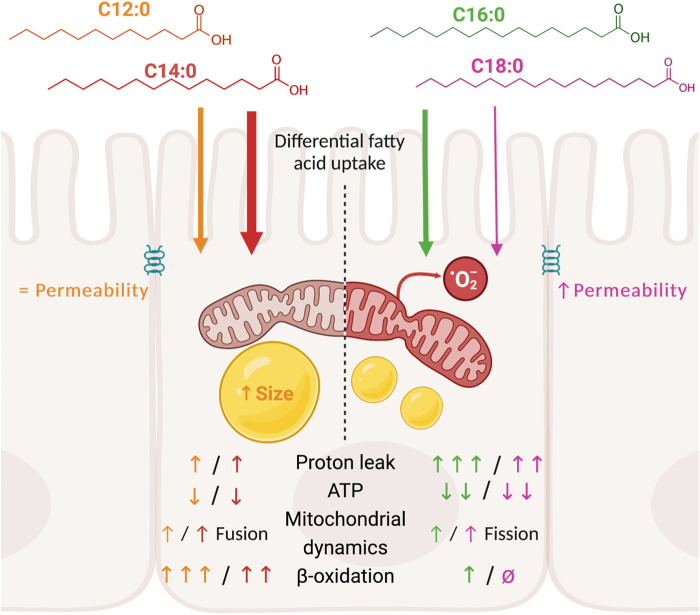

## Introduction

The intestinal epithelial barrier consists in a monolayer of intestinal epithelial cells (IECs) joined by intercellular tight junctions which regulate paracellular permeability. The maintenance of a tight barrier relies notably on the mitochondrial function of IECs, which generates energy through oxidative phosphorylation (OXPHOS) to ensure tight junction protein regulation through phosphorylation (Tsukamoto and Nigam, 1999). Hence, induction of metabolic stress by dinitrophenol *in vitro* in IEC lines and *in vivo* in rats resulted in increased internalization of the non-pathogenic *Escherichia coli* (Nazli et al., 2004), oxidative stress (Wang et al., 2014), and internalization of some tight junction proteins from the plasma membrane toward the cytosol (JanssenDuijghuijsen et al., 2017). In enterocytes, energy is mainly provided through the oxidation of glutamine and, to a lesser extent, of glucose, but IECs are also able to β-oxidize dietary fatty acids to produce ATP (Alpers, 2000). For example, IECs from high-fat diet (HFD)-fed rodents are characterized by not only elevated β-oxidative activity (Kondo et al., 2006) but also oxidative stress (Qiao et al., 2013; Gil-Cardoso et al., 2017). Indeed, enhanced fatty acid β-oxidation has been linked to increased reactive oxygen species (ROS) production in the liver (Seifert et al., 2010; Kakimoto et al., 2015). Such increase in ROS may potentiate alterations of mitochondria, through lipid peroxidation, attack on OXPHOS complexes, and/or mitochondrial DNA damages (Kirkinezos and Moraes, 2001). Moreover, ROS may alter tight junctions, notably *via* the redistribution of tight junction proteins toward the intracellular compartment (Rao, 2008) or the phosphorylation of occludin (Gangwar et al., 2017). To date, the effects of dietary fatty acids on the mitochondrial function of IECs and the associated consequences on gut permeability remain poorly described [for a review, see the work of Guerbette et al. (2022)]. A few studies reported that a 24-h treatment of colonic epithelial cell lines (HCT-116, NCI-H716, and Caco-2) with palmitic acid (C16:0) at concentrations ranging from 100 µM to 2.5 mM induced oxidative stress, disrupted the mitochondrial network, decreased the mitochondrial membrane potential, and altered IEC bioenergetics (Lee et al., 2020; Li and Li, 2020; Yoo et al., 2021). Moreover, several studies reported that a 24-h treatment of Caco-2 cells with C16:0 increased the electrical conductance of the monolayer, indicating enhanced paracellular flux through tight junctions, and enhanced the passage of FITC-dextran across the monolayer due to the relocalization of some tight junction proteins and/or a decrease in their expression (Ghezzal et al., 2020; Gori et al., 2020). However, although C16:0 is the main dietary saturated fatty acid (30–38 g/day) because it is universally found in natural fats (Katan et al., 1994), the effects of other dietary saturated fatty acids on the mitochondrial function and permeability of IECs are not well characterized. We, thus, evaluated the impact of a chronic treatment method with the main saturated fatty acids found in human diet, *i.e.*, C12:0, C14:0, C16:0, and C18:0, on mitochondrial function, ROS production, and epithelial permeability in the *in vitro* porcine enterocyte model IPEC-J2. In addition, recent works showed that a 2-day treatment with C12:0 (Yang et al., 2020b) or C18:0 (Yang et al., 2020a), from 100 µM to 250 μM, did not alter cell viability. Yet, millimolar concentrations of either fatty acid induced IPEC-J2 apoptosis. Consequently, IPEC-J2 cells were treated at the intermediate concentration of 250 µM of each fatty acid since it may favor enterocyte differentiation without altering cell viability. Although the lipid metabolism of IPEC-J2 is not fully characterized, these cells are considered a suitable model of small intestinal epithelial cells. Since they are neither cancerous, unlike most *in vitro* models of IEC, nor transformed (Vergauwen, 2015), IPEC-J2 cells exhibit a predominantly OXPHOS-dependent oxidative metabolism of glutamine (Bernardini et al., 2021), similar to healthy enterocytes *in vivo*. In addition, IPEC-J2 cells spontaneously differentiate within a few days to form a polarized epithelium with high transepithelial electrical resistance (TEER) (Vergauwen, 2015), making them a relevant model for studying small intestinal barrier integrity.

## Materials and methods

### IPEC-J2 culture

IPEC-J2 cells were maintained in Dulbecco’s modified Eagle medium (DMEM) with GlutaMAX (31966-021, Gibco) supplemented with 10% fetal bovine serum (HyClone, SH30066.03) and 1% penicillin/streptomycin (100 U/mL/100 𝜇g/mL) in a humidified atmosphere of 5% CO_2_ at 37°C. The medium was changed every 2–3 days. The cells were passaged once a week and seeded at a density of 100,000 cells/cm^2^. Once they reached confluence, IPEC-J2 cells were treated for 3 days with 250 µM of either C12:0 (Sigma-Aldrich, L4250), C14:0 (Sigma-Aldrich, M3128), C16:0 (Sigma-Aldrich, P5585), or C18:0 (Sigma-Aldrich, S4751) diluted in dimethylsulfoxide (DMSO; Sigma-Aldrich, D8418) to a final concentration of 0.6%. The control condition (CTRL) corresponds to the IPEC-J2 treated for 3 days with 0.6% DMSO. The treatment was changed every day.

### Neutral lipid staining

Nile red staining was performed after cells were fixed into a 4% paraformaldehyde solution for 30 min. The Nile red powder (Thermo Fisher Scientific, N1142) was first diluted at 1 mg/mL into DMSO. Cells were incubated for 30 min at room temperature in a 100 μg/mL Nile red solution, diluted in PBS. After incubation, the cells were washed with PBS, and fluorescence was read with excitation/emission wavelengths of 520 nm/590 nm using a BMG Labtech POLARstar Omega microplate reader. The results were normalized using Hoechst 33258 (Thermo Fisher Scientific, H3569) fluorescence intensity, which stains double-strain DNA, read at 355 nm/460 nm.

### Cell viability and toxicity assays

Cell viability after treatment was measured by the methylthiazolyldiphenyl-tetrazolium bromide (MTT) assay (M5655, Sigma-Aldrich) by adding 0.5 mg/mL MTT for 3 h in an incubator at 37°C and 5% CO_2_. The cells were lysed using 0.1 M isopropanol/HCl +2% Triton-X100 for 1 h under gentle shaking at room temperature. Absorbance was finally read at 570 nm using a microplate reader (BMG Labtech, POLARstar Omega). Cytotoxicity was evaluated by measuring the activity of the lactate dehydrogenase (LDH) released in the supernatants of cells after the last 24 h of treatment according to the manufacturer’s instructions from the cytotoxicity detection kit (11644793001, Sigma-Aldrich).

### Fatty acid uptake and export in triglycerides

IPEC-J2 cells were grown on 6-well plates (140675, Thermo Fisher Scientific). After 3 days, the cells were treated for 3 h with 250 µM of C12:0, C14:0, C16:0, or C18:0 containing either 5 μCi/μmol of [1–^14^C]C12:0, [1–^14^C]C14:0, [1–^14^C]C16:0, or [1–^14^C]C18:0 at 37°C and 5% CO_2_ and then washed twice in PBS. Radioactivity in the conditioned media and cells diluted in 10 mL of scintillation liquid was then counted using a liquid scintillation analyzer (Perkin Elmer, Tri-Carb B28100). To assay the export of radiolabeled fatty acids as triglycerides in secreted chylomicrons, the medium was collected after 3 h of treatment, and total lipids were extracted using 1 mL of HCl and 4 mL of a mix of hexane/isopropanol (3:2; vol:vol). After a 1000xg centrifugation for 5 min, the organic phase was collected. After evaporation of the organic phase under nitrogen flow, total lipids were resuspended in 200 µL of chloroform and were deposited on a thin silica layer to separate the classes of lipids by using a mixture of hexane/diethylether/acetic acid (80:20:0.5 vol:vol:vol). Triglycerides were collected from silica using an ether/hexane/NaCl (3:4:3 vol:vol:vol) solution and centrifuged for 5 min at 1000xg. The upper phase was finally collected and transferred into 10 mL of scintillation liquid for radioactivity counting.

### Fatty acid quantification from the total lipid content in IPEC-J2

IPEC-J2 cells were grown on a 60-mm diameter Petri dish and were treated for 3 days with either 250 µM of C12:0, C14:0, C16:0, or C18:0. The cells were then washed in PBS, collected into 3 mL of PBS, and centrifuged at 1000xg for 5 min to eliminate residual fatty acids. The pellet was resuspended in 1 mL of PBS with 900 µL used for lipid assay and 100 µL for protein quantification. 15 μg of C13:0 was added to each sample as an internal standard for quantification. Lipid extraction was performed by adding 4 mL of a mix of 3:2 (vol:vol) hexane/isopropanol and 1 mL of HCl to acidify. After 15 min of agitation at room temperature, the samples were centrifugated at 1000xg for 10 min, and the upper phase containing lipids was transferred to a new tube containing 150 mM of NaCl for rinsing. After a vigorous shake and centrifugation at 1000xg for 3 min, the hexane was collected and evaporated with nitrogen flow at 55°C. To saponify lipids, 500 µM of NaOH was added for 20 min at 70°C. Methylation was then performed by adding 1.5 M BF_3_ in methanol for 20 min at 70°C. 150 mM of NaCl and pentane were added in each tube in order to isolate fatty acid methyl esters (FAME) in the upper phase, which was further evaporated as previously described. Finally, FAME were resuspended in 50 µL of hexane. The total concentration of FAME was analyzed by injecting 1 µL of each sample in an Agilent 7890N gas chromatograph coupled to a 5975C mass spectrometry detector (Agilent), as previously described (Drouin et al., 2019). The quantified fatty acids were normalized by mg of proteins using the BCA Protein Assay Kit (23225, Thermo Fisher Scientific) and expressed in µg/mg of proteins.

### Measurement of TEER

TEER was measured after the cells were seeded onto transparent plastic inserts for 24-well plates (353504, Falcon) with 0.4-μm pores and treated with the different fatty acids as described above. The TEER was monitored every 30 min for 3 days using the CellZscope+ (nanoAnalytics).

### Gene expression

Cells were lysed in RA1 buffer, and the total RNA was extracted according to the manufacturer’s instructions (740955.250, Macherey-Nagel). After concentration and quality assessments using a NanoDrop (NanoDrop^®^1000, Thermo Fisher), RNAs were diluted in ultrapure water (10977035, Thermo Fisher) to a final concentration of 100 ng/μL. Reverse transcription into cDNA was performed using the High-Capacity cDNA Reverse Transcription Kit (4388950, Applied Biosystems), and cDNA was diluted to a final concentration of 5 ng/μL. Quantitative PCR was performed with the PowerSYBR Green PCR Master Mix (4309155, Applied Biosystems). Gene expression was analyzed according to the 2^−ΔΔCT^ method using TATA box-binding protein (*Tbp*) as the housekeeping gene. All the primers used had an efficiency level above 85%, and the sequences are available in Supplementary Table S1.

### Bioenergetic analysis

Bioenergetic analyses were performed using the Seahorse XFe24 Analyzer. Cells were seeded in a 24-well plate for Seahorse (100777-004, Agilent) and treated for 3 days as described above. The day before the experiment, the sensor cartridge from the Xfe24 FluxPak (102,340-100, Agilent) was immersed in the Seahorse XF calibrant solution (100840-000, Agilent) and incubated at 37°C without CO_2_ overnight. On the day of experiment, Seahorse medium (102353-100, Agilent) was prepared by adding pyruvate (1 mM, 103578-100, Agilent), L-glutamine (2 mM, 103579-100, Agilent), and glucose (10 mM, Agilent, 103577-100), and the pH was adjusted to 7.4. One hour before starting the analysis, the cells were washed with the complete Seahorse medium and incubated for 45 min at 37°C in an incubator without CO_2_ while the analyzer was calibrating. Mitochondrial function analysis was performed using the Cell Mito Stress Test Kit (103015-100, Agilent). Measurements were carried out in cycles of 3 min mix, 2 min wait, and 3 min measure before and after oligomycin (2 µM), carbonyl cyanide-4-phenylhydrazone (FCCP; 2 µM), and antimycin A/rotenone (0.5 µM) injections. Three measurement cycles of oxygen consumption rate (OCR) were performed at the basal state, before any injection, and at the metabolic stress state, simulated by the FCCP injection. Once the assay was completed, cells were fixed and stained with Hoechst for normalization.

### Mitochondrial network analysis

Mitochondrial network analysis was performed *via* immunostaining of the translocase of outer mitochondrial membrane 20 (TOMM20). IPEC-J2 cells were seeded on Lab-Tek chamber slides (Thermo Fisher, 177445) and treated with 250 µM of each fatty acid for 3 days. They were then fixed with a 4% paraformaldehyde solution for 20 min at room temperature and rinsed with wash buffer (0.1% BSA in 1X PBS). The cells were then incubated for 45 min at room temperature in a blocking solution (PBS, FBS 10%, and Triton X-100 0.3%) and incubated overnight at 4°C with an anti-TOMM20 (Abcam, ab186735) primary antibody diluted at 1:250 in a dilution buffer (PBS, BSA 1%, FBS 1%, Triton X-100 0.3%, and sodium azide 0.01%). After rinsing, the cells were incubated for 1 h at room temperature with an anti-rabbit Alexa Fluor 594 secondary antibody (R37119, Thermo Fisher), diluted at 1:200 in the dilution buffer. Finally, IPEC-J2 cells were rinsed and stained with Hoechst. Mitochondrial network analysis was performed by adapting a protocol from the work of Valente et al. (2017), which consisted in superimposing 0.3-µm sections from the confocal image Z-stack, taken using a ZEISS LSM 980 with Airyscan 2 confocal microscope, and skeletonizing the TOMM20 signal on ImageJ to determine branch lengths.

### ATP production

IPEC-J2 cells were treated for 3 days with each fatty acid as described above, and ATP production at the end of the treatment was measured by bioluminescence using a luminescent ATP detection kit (ab113849, Abcam) by following the manufacturer’s instructions.

### Western blot

After 3 days of treatment, IPEC-J2 cells were rinsed twice in PBS, lysed in RIPA buffer (R0278, Sigma-Aldrich), and sonicated before protein quantification and denaturation in Laemmli buffer. 14 µg of total protein lysates were separated in 4%–12% Bis-tris SDS-PAGE gel and transferred onto PVDF membranes. The membranes were saturated for 2 h at room temperature in a solution of 5% bovine serum albumin (BSA) in tris-buffered saline containing 0.1% Tween 20 (TBST). Proteins were then immunoblotted overnight (4°C) with primary antibodies against citrate synthase (CS; ab96600, Abcam), TOMM20 (ab186735, Abcam), or with Heat shock cognate 71 kDa protein (HSC70; sc7298, Santa-Cruz). After three TBST washes, blots were incubated in the appropriate secondary antibodies linked to HRP for 2 h at RT in a 5% BSA/TBST solution, and enhanced chemiluminescence (34076, Thermo Fisher) revealed protein bands. The mean gray value of each band was measured using ImageJ and normalized by the HSC70 signal.

### ROS detection

The MitoSOX probe (M36008, Invitrogen) was used to detect mitochondrial superoxide anions. Cells were incubated at 37°C, 5% CO_2_ for 10 min with 5 µM MitoSOX diluted in Hank’s Balanced Salt Solution (HBSS; 14175-095, Thermo Fisher). After a wash in HBSS, fluorescence was measured using a BMG Labtech POLARstar Omega microplate reader with excitation/emission wavelengths of 520 nm/590 nm. Data were normalized by the Hoechst fluorescence intensity.

### Measurement of β-oxidation products

Measurement of acid-soluble metabolites (ASM) and CO_2_ from β-oxidation of [1–^14^C]C12:0, [1–^14^C]C14:0, [1–^14^C]C16:0, or [1–^14^C]C18:0 was performed as described by Rioux et al. (2003). In brief, cells were grown on a 60-mm diameter Petri dish. After 3 days of culture in CTRL medium, IPEC-J2 cells were either treated with 250 µM of C12:0, C14:0, C16:0, or C18:0, containing 5 μCi/μmol of radiolabeled fatty acid [1–^14^C]C12:0, [1–^14^C]C14:0, [1–^14^C]C16:0, or [1–^14^C]C18:0, respectively. To measure ^14^CO_2_, the IPEC-J2 and medium were collected into glass tubes containing 250 μL of 7 M HClO_4_ and sealed with a rubber cap containing a plastic well. Benzethonium hydroxide (B2156, Sigma) was introduced into the suspended plastic well by piercing the rubber cap with a syringe. Tubes were incubated at 37°C for 3 h in order to trap ^14^CO_2_. The precipitated ^14^CO_2_ was resuspended into a Na_2_CO_3_ and BaOH_2_ solution (1.2:10; vol:vol) and counted using a liquid scintillation analyzer. Measurement of ASM from β-oxidation was performed by transferring the IPEC-J2 and medium into tubes containing 250 μL of 7 M HClO_4_, maintained on ice for 15 min, and centrifuged at 2500xg for 10 min at 4°C. In order to remove any residual ^14^C-labeled fatty acids, the collected supernatants were washed three times with hexane, and radioactivity was finally counted.

### Statistical analysis

Data are shown as the mean ± standard error of the mean of at least three independent cell passages. Statistical analyses on experimental data, including gene expression analysis using the 2^−ΔΔCT^ method, were performed using unpaired Student’s t-test in GraphPad Prism 8. Significant results are shown with *p < 0.05, **p < 0.01, and ***p < 0.005 vs. CTRL, ^α^P < 0.05, ^αα^P < 0.01, and ^ααα^P < 0.005 vs. C12:0; ^β^P < 0.05, ^ββ^P < 0.01, and ^βββ^P < 0.005 vs. C14:0; and ^γ^P < 0.05, ^γγ^P < 0.01, and ^γγγ^P < 0.005 vs. C16:0.

## Results

### Treatment of IPEC-J2 with 250 µM of C12:0, C14:0, C16:0, or C18:0 for 3 days did not alter cell viability

Cell viability and death were evaluated by measuring the MTT signal and the activity of the LDH released in the cell culture supernatant, respectively. Treatment with 250 µM of C12:0 or C14:0 did not alter MTT signal intensity (CTRL: 100% ± 0.0%, C12:0: 94.2% ± 4.3%, and C14:0: 96.1% ± 11.8%), while it significantly increased with C16:0 and C18:0 (CTRL: 100% ± 0.0% vs. C16:0: 175.5% ± 22.8%, *p* = 0.008 and CTRL vs. C18:0: 152.3 ± 15.1, *p* = 0.006). None of the fatty acids significantly increased LDH activity in the cell supernatant compared to CTRL (CTRL: 100% ± 0.0%; C12:0: 89.5% ± 13.2%; C14:0: 132.0 ± 20.3; C16:0: 126.8% ± 12.5%; and C18:0: 142.7 ± 30.1). Taken together, these data indicate that none of the fatty acids neither diminished cell viability nor induced death after 3 days of treatment at 250 µM.

### C12:0 and C14:0 induced greater lipid storage in IPEC-J2 compared to C16:0 and C18:0

**FIGURE 1:**
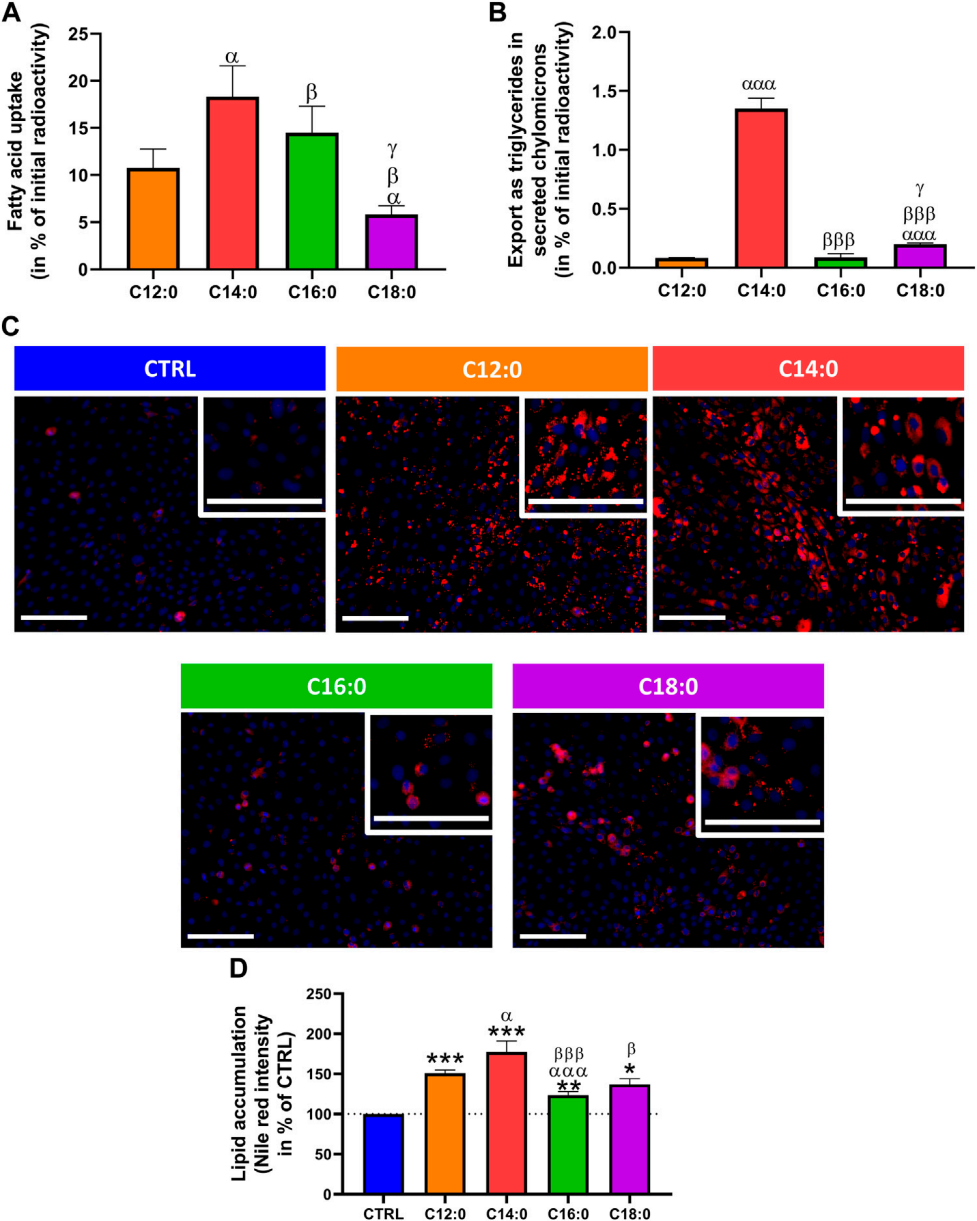
Fatty acid absorption and storage in IPEC-J2. **(A)** Fatty acid uptake in IPEC-J2 and **(B)** export as triglycerides in secreted chylomicrons after 3 h of treatment with either [1–^14^C]C12:0, [1–^14^C]C14:0, [1–^14^C]C16:0, or [1–^14^C]C18:0, without prior chronic treatment. **(C)** Representative images of neutral lipid staining (Nile red in red) in IPEC-J2 counterstained with Hoechst (blue) after 3 days of C12:0, C14:0, C16:0, or C18:0 treatments at 250 µM. The scale bar represents 100 µm. **(D)** Quantification of the Nile red staining normalized by the Hoechst fluorescence intensity and expressed in percentage of CTRL. N = 3–6 for each condition from independent experiments. Significant differences are represented as *p < 0.05, **p < 0.01, and ***p < 0.001 vs. CTRL; ^α^P < 0.05 and ^ααα^P<0.001 vs. C12:0; ^β^P < 0.05 and ^βββ^P<0.001 vs. C14:0; and ^γ^P < 0.05 vs. C16:0.

The ability of IPEC-J2 cells to differentially absorb, metabolize, and export saturated fatty acids as a function of their chain length was first evaluated by measuring the radiolabeled fatty acids taken up and those exported as triglycerides in secreted chylomicrons in the cell culture medium after a 3-h treatment with either 250 µM of C12:0, C14:0, C16:0, or C18:0, without prior chronic treatment. 10% of initially incubated C12:0 entered the enterocytes, whereas the uptake of C14:0 was 8% more than that of C12:0 (*p* = 0.02). The uptake of C16:0 was nearly 4% lower than that of C14:0 (*p* = 0.01) and was 9% more than that of C18:0 (*p* = 0.02) after 3 h (Figure 1A). As for the export in secreted triglycerides, C14:0 was the most exported fatty acid into the cell culture media, representing an export nearly 17 times greater than that of C12:0 (*p* = 0.0001), followed by C18:0, whose export is 2.5 greater than that of C12:0 (*p* = 0.0004 vs. C18:0) and C16:0 (*p* = 0.03 vs. C18:0), which were barely exported in triglycerides (Figure 1B). After 3 days of chronic treatment with the different fatty acids, lipid storage in IPEC-J2 was evaluated by staining neutral lipids (Figure 1C). Quantification of Nile red staining, normalized by Hoechst, revealed that C12:0 and C14:0 induced significant lipid storage in IPEC-J2, which was increased by 50.9% ± 4.0% (*p* < 0.001 vs. CTRL) and 77.5% ± 13.5% (*p* < 0.0005 vs. CTRL), respectively, compared to CTRL (Figure 1D). C16:0 and C18:0 induced also an increase in lipid storage, with 23.4% ± 4.7% (*p* = 0.005 vs. CTRL) and 37.1% ± 7.0% (*p* = 0.004 vs. CTRL) increases compared to CTRL, respectively, which were lower than those with C12:0 and C14:0, although the difference between C12:0 and C18:0 was not significant (*p* = 0.08) (Figure 1D).

### IPEC-J2 differentially converted saturated fatty acids into longer-chain fatty acids

**FIGURE 2:**
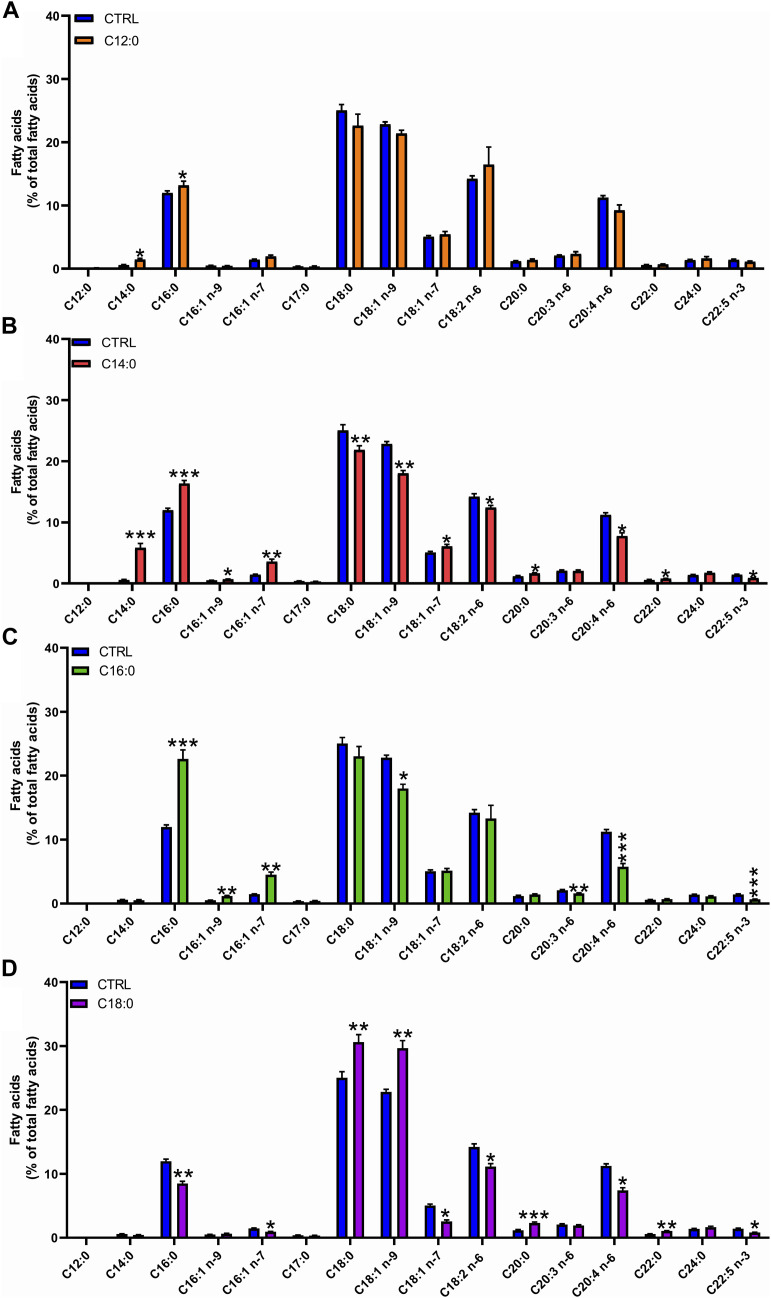
Fatty acid composition of total lipids in response to fatty acid treatment. IPEC-J2 cells were treated for 3 days with 250 µM of C12:0, C14:0, C16:0, or C18:0. Fatty acid composition of total lipids was analyzed by GC–MS and expressed in the percentage of the total lipid content after **(A)** C12:0, **(B)** C14:0, **(C)** C16:0, or **(D)** C18:0 treatments. Data are represented as mean ± SEM. Significant differences are represented as *p < 0.05, **p < 0.01, and ***p < 0.005 vs. CTRL.

To determine the metabolic fate of fatty acids, analysis of the fatty acid composition of total lipids was performed after 3 days of treatment with 250 µM of either C12:0, C14:0, C16:0, or C18:0. C14:0 and C12:0 treatments induced the production of C16:0, indicating an elongation of C12:0 and C14:0 into C16:0. Treatment with C14:0 also resulted in the production of C16:1 (n-7), indicating that C16:0, obtained from the elongation of C14:0, was further Δ9-desaturated in C16:1 (n-7) (Figures 2A, B). The higher amount of C16:0 may also be partly explained by the β-oxidation of C12:0 and C14:0 producing acetyl-CoA, followed by the *de novo* lipogenesis of C16:0. Treatment with C14:0 also induced the production of C18:1 (n-7) (further elongation of C16:1 n-7). High proportions of C16:0 and C16:1 (n-7) were also measured in IPEC-J2 after C16:0 treatment, indicating a Δ9-desaturation of C16:0 into C16:1 (n-7) as well (Figure 2C). The higher proportion of C16:1 (n-9) measured after C16:0 treatment is likely to arise from the partial peroxisomal β-oxidation of C18:1 (n-9). Treatment with C18:0 increased the production of C18:1 (n-9), indicating the Δ9-desaturation of C18:0 into C18:1 (n-9). Treatment with C18:0 also increased the concentration of C20:0 and C22:0, indicating an elongation of C18:0 into these longer chains (Figure 2D).

### C16:0 and C18:0 increased epithelial paracellular permeability after 24 h of treatment

**FIGURE 3:**
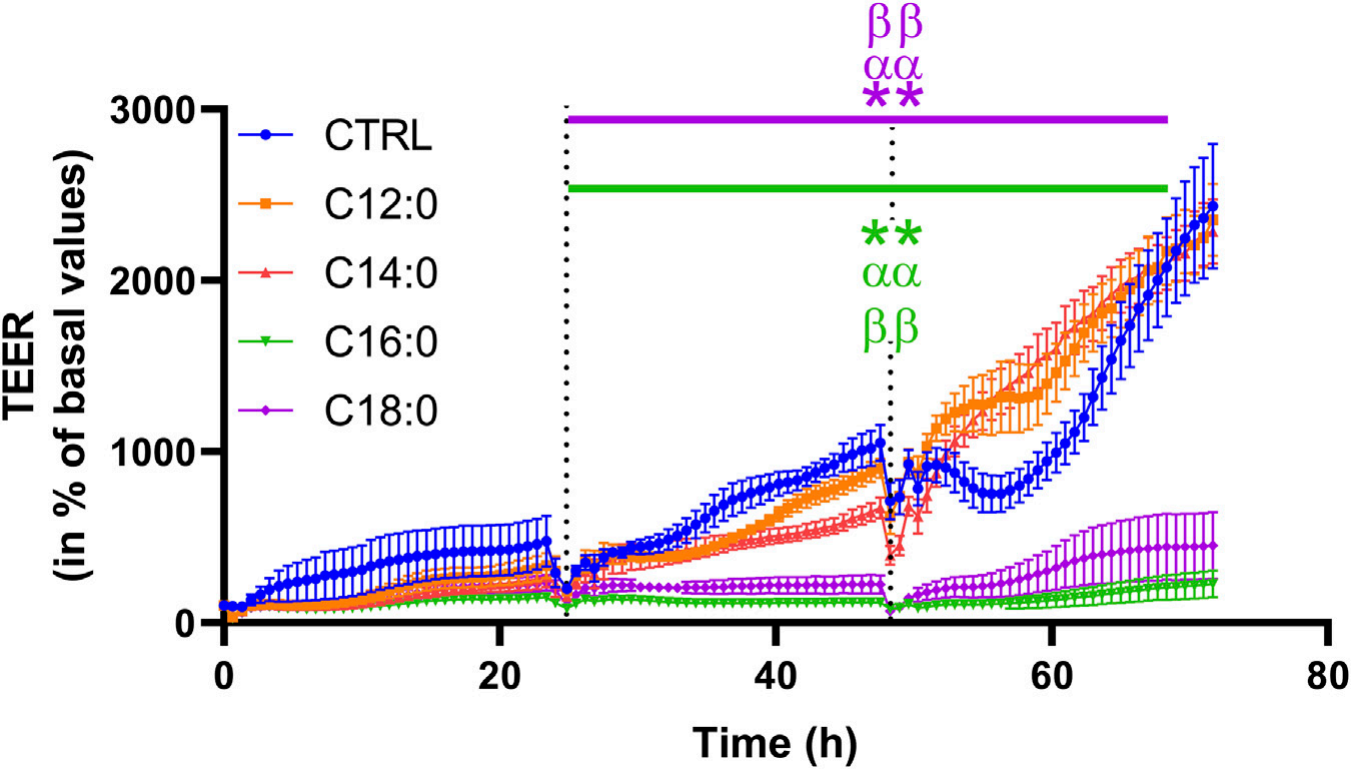
Transepithelial electrical resistance over time of IPEC-J2 in response to fatty acid treatments. The TEER was measured during the 3 days of C12:0, C14:0, C16:0, or C18:0 (250 µM each) treatment and represented over time (percentage of initial values for each condition). Dashed lines correspond to fatty acid addition to the media. N = 3 for each condition from independent experiments. The data are presented as mean ± SEM. Significant differences are represented as **p < 0.01 vs. CTRL, ^αα^P < 0.01 vs. C12:0, and ^ββ^P < 0.01 vs. C14:0.

To evaluate the impact of 3-day chronic fatty acid treatment on paracellular permeability over time, IPEC-J2 TEER was measured continuously during the 3 days with 250 µM of C12:0, C14:0, C16:0, or C18:0. While the treatments with C12:0 and C14:0 did not modify TEER compared to the CTRL condition, C16:0 and C18:0 significantly decreased it starting after 24 h and until the end of the treatment, suggesting increased paracellular flux (Figure 3). Since the increase in paracellular permeability could originate from the diminished expression of tight junction proteins, the expression of genes encoding some tight junction proteins (*Cldn1*, *Cldn2*, *Tjp1*, *Ocln*, and *Cldn7*) was measured. Gene expression was altered in response to neither C16:0 nor C18:0 treatment compared to CTRL (Table 1).

**TABLE 1:** Relative mRNA expression of genes encoding tight junction proteins.

	CTRL	C12:0	C14:0	C16:0	C18:0
***Cldn1***	1.0 ± 0.1	0.9 ± 0.2	0.8 ± 0.2	0.9 ± 0.3	0.8 ± 0.3
***Cldn2***	1.1 ± 0.6	0.7 ± 0.7	0.8 ± 0.5	1.3 ± 1.3	1.1 ± 1.7
***Tjp1***	1.0 ± 0.1	0.9 ± 0.2	1.1 ± 0.2	1.1 ± 0.4	1.2 ± 0.5
*Ocln*	1.0 ± 0.2	1.0 ± 0.2	0.9 ± 0.4	0.8 ± 0.3	0.8 ± 0.4
***Cldn7***	1.0 ± 0.1	1.0 ± 0.3	0.9 ± 0.3	0.9 ± 0.4	0.9 ± 0.6

Data are mean ± SEM.

### C16:0 and C18:0 induced a severe decrease in mitochondrial ATP-associated respiration and ATP production compared to C12:0 and C14:0

**FIGURE 4:**
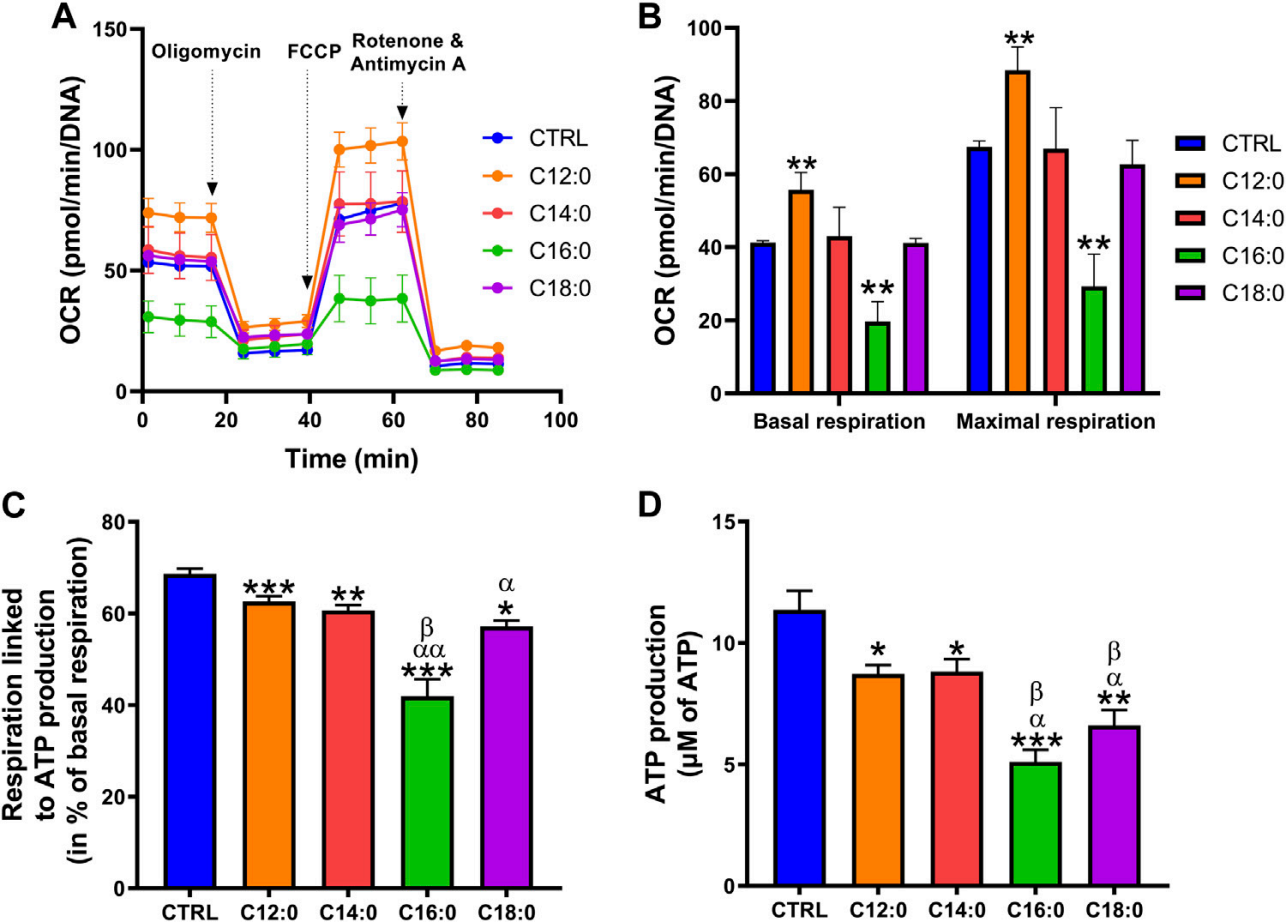
Differential alterations of mitochondrial bioenergetics after fatty acid treatments. IPEC-J2 cells were treated for 3 days with 250 µM of C12:0, C14:0, C16:0, or C18:0. **(A)** The OCR of IPEC-J2 cells after 3 days of treatment with the fatty acids and **(B)** basal and maximal respirations normalized by the Hoechst fluorescence intensity. **(C)** Part of the basal respiration linked to ATP production determined by the Seahorse analysis. **(D)** Measurement of ATP production. N = 3–8 for CTRL, C12:0, and C16:0; N = 3–5 for C14:0; and N = 3 for C18:0 from independent experiments. Data are represented as mean ± SEM. Significant differences are represented as *p < 0.05, **p < 0.01, and ***p < 0.001 vs. CTRL, ^α^P < 0.05; ^αα^P<0.01 vs. C12:0, and ^β^P < 0.05 vs. C14:0.

To assess the impact of each fatty acid on the mitochondrial function of IPEC-J2, OCR and bioenergetic parameters were measured using the Seahorse technology (Figure 4A and Supplementary Figure S1). Analysis of OCR revealed that treatment with C12:0 increased basal and maximal respirations of IPEC-J2, whereas they were diminished by half in response to C16:0. Neither C14:0 nor C18:0 modified these two parameters (Figure 4B). Analysis of the bioenergetic parameters of IPEC-J2 revealed that all fatty acids decreased the part of basal respiration used for ATP production and, more particularly, C16:0 and C18:0, which diminished it by 40% (*p* < 0.0001) and 17% (*p* = 0.0004), respectively, while C12:0 and C14:0 diminished it by less than 10% (C12:0 vs. CTRL: *p* = 0.003; C14:0 vs. CTRL: *p* = 0.0008) (Figure 4C). In connection with the respiration used to produce ATP, the overall ATP content of IPEC-J2 after 3 days of treatment was diminished by all the saturated fatty acids from 25% after a C12:0 or C14:0 treatment to 50% with C16:0 or C18:0 (Figure 4D). However, no significant change in the expression of genes encoding subunits from each of the five mitochondrial complexes was measured (Supplementary Table S2).

### Fatty acids differentially modulated the mitochondrial network without modifying mitochondrial mass

**FIGURE 5:**
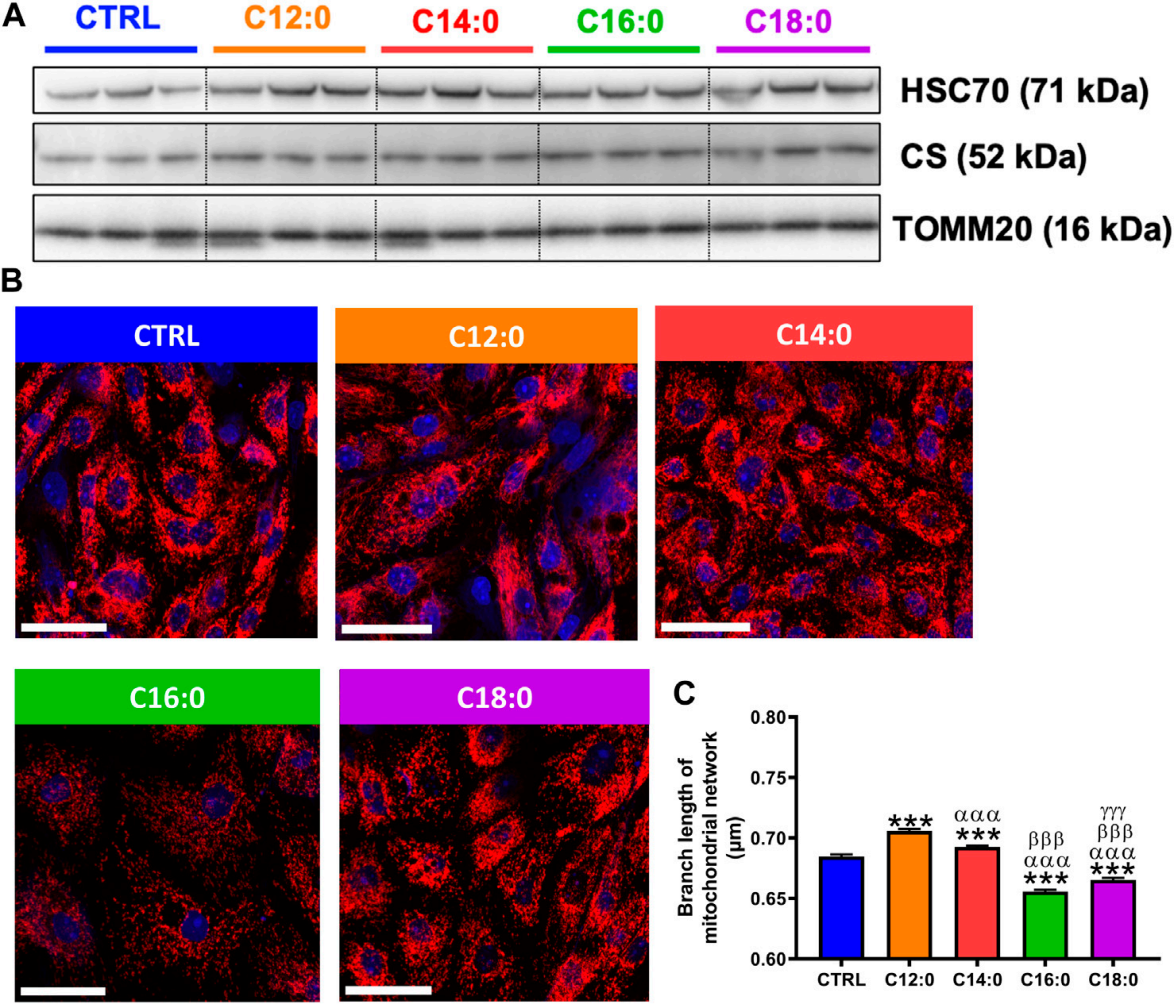
Mitochondrial dynamics in response to fatty acid treatments. IPEC-J2 cells were treated for 3 days with 250 µM of C12:0, C14:0, C16:0, or C18:0. **(A)** Protein expression of TOMM20 and CS by Western blot. **(B)** Representative confocal images of TOMM20 immunostaining (red) in IPEC-J2 counterstained with Hoechst (blue). The scale bar represents 50 µm. **(C)** Branch length analysis of the mitochondrial network from confocal images. N = 3 from independent experiments. Data are represented as mean ± SEM. Significant differences are represented as ***p < 0.001 vs. CTRL; ^ααα^P < 0.001 vs. C12:0; ^βββ^P < 0.001 vs. C14:0; and ^γγγ^P < 0.001 vs. C16:0.

Since mitochondrial bioenergetics is closely related to mitochondrial dynamics, mitochondrial mass was first determined by measuring the protein expression of TOMM20 and CS by Western blot. No significant change in the expression of TOMM20 or CS was measured in response to fatty acid treatment, indicating unaltered mitochondrial mass after treatments (Figure 5A). Then, analysis of the mitochondrial network was performed in response to fatty acids using TOMM20 immunocytostaining (Figure 5B). Mitochondrial network analysis performed on the confocal images indicated that C16:0 and C18:0 induced a decrease in mitochondrial branch length (Figure 5C). On the contrary, both C12:0 and C14:0 increased the mitochondrial network branch length. Taken together, these data suggest that fatty acids did not modify mitochondrial mass but induced remodeling of the mitochondrial network toward fusion in response to C12:0 and C14:0 and fragmentation after C16:0 and C18:0 treatments.

### C16:0 or C18:0 treatment elevated mitochondrial ROS production

**FIGURE 6:**
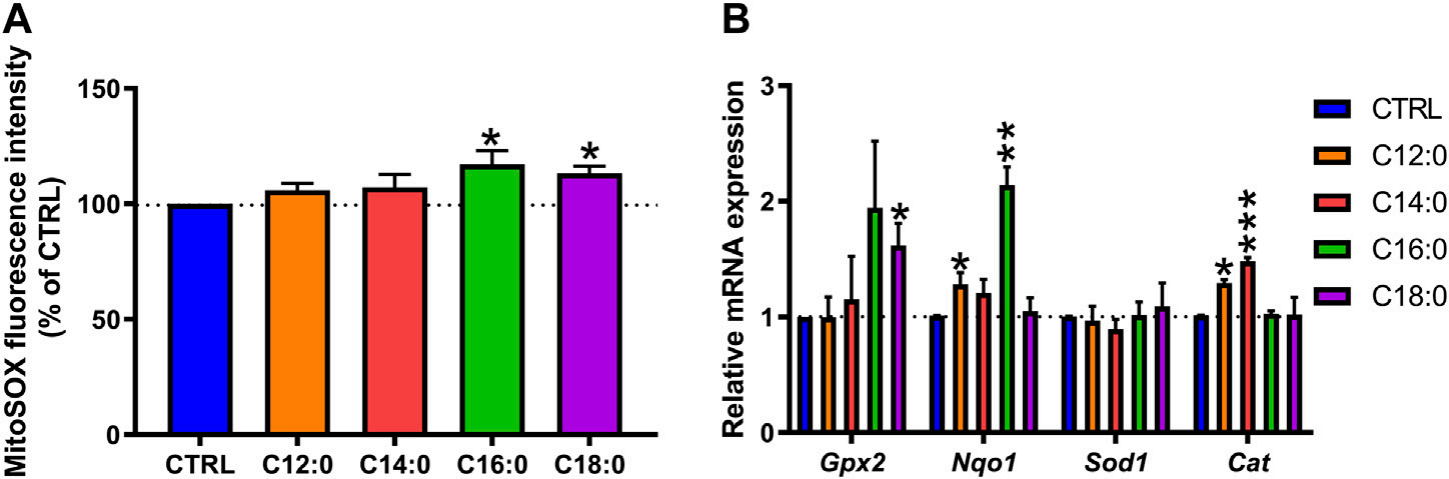
Mitochondrial reactive oxygen species detection and induction of the antioxidant machinery after fatty acid treatments. IPEC-J2 cells were treated for 3 days with 250 µM of C12:0, C14:0, C16:0, or C18:0. **(A)** Detection of mitochondrial ROS by MitoSOX dye labeling expressed in the percentage of CTRL after normalization by the Hoechst fluorescence intensity. **(B)** Relative mRNA expression of genes encoding antioxidant enzymes. N = 3 for each condition from independent experiments. Data are represented as mean ± SEM. Significant differences are represented as *p < 0.05, **p < 0.01, and ***p < 0.001 vs. CTRL.

Given the differential alterations of enterocyte metabolism induced by the fatty acid treatments, mitochondrial ROS production was measured at the basal state. C12:0 or C14:0 treatment did not modify the production of mitochondrial ROS (Figure 6A) and induced the expression of the gene encoding catalase (*p* < 0.01), which converts H_2_O_2_ into H_2_O and O_2_ (Figure 6B). Increased *Cat* expression associated with elevated non-mitochondrial respiration (Supplementary Figure S1) induced by C12:0 (*p* < 0.01) suggests increased peroxisomal activity or β-oxidation. On the other hand, treatments with C16:0 (*p* = 0.04) or C18:0 (*p* = 0.01) induced a greater generation of mitochondrial ROS compared to CTRL (Figure 6A). Moreover, C16:0 treatment increased the expression of the gene that encodes NQO1 (*p* < 0.01), which scavenges ROS through quinone recycling, while C18:0 treatment increased that of GPX2 (*p* = 0.03), which catalyzes the reduction of cytosolic H_2_O_2_ into H_2_O and O_2_ (Figure 6B).

### C16:0 and C18:0 induced greater OXPHOS uncoupling than C12:0 and C14:0 independent of their β-oxidation

**FIGURE 7:**
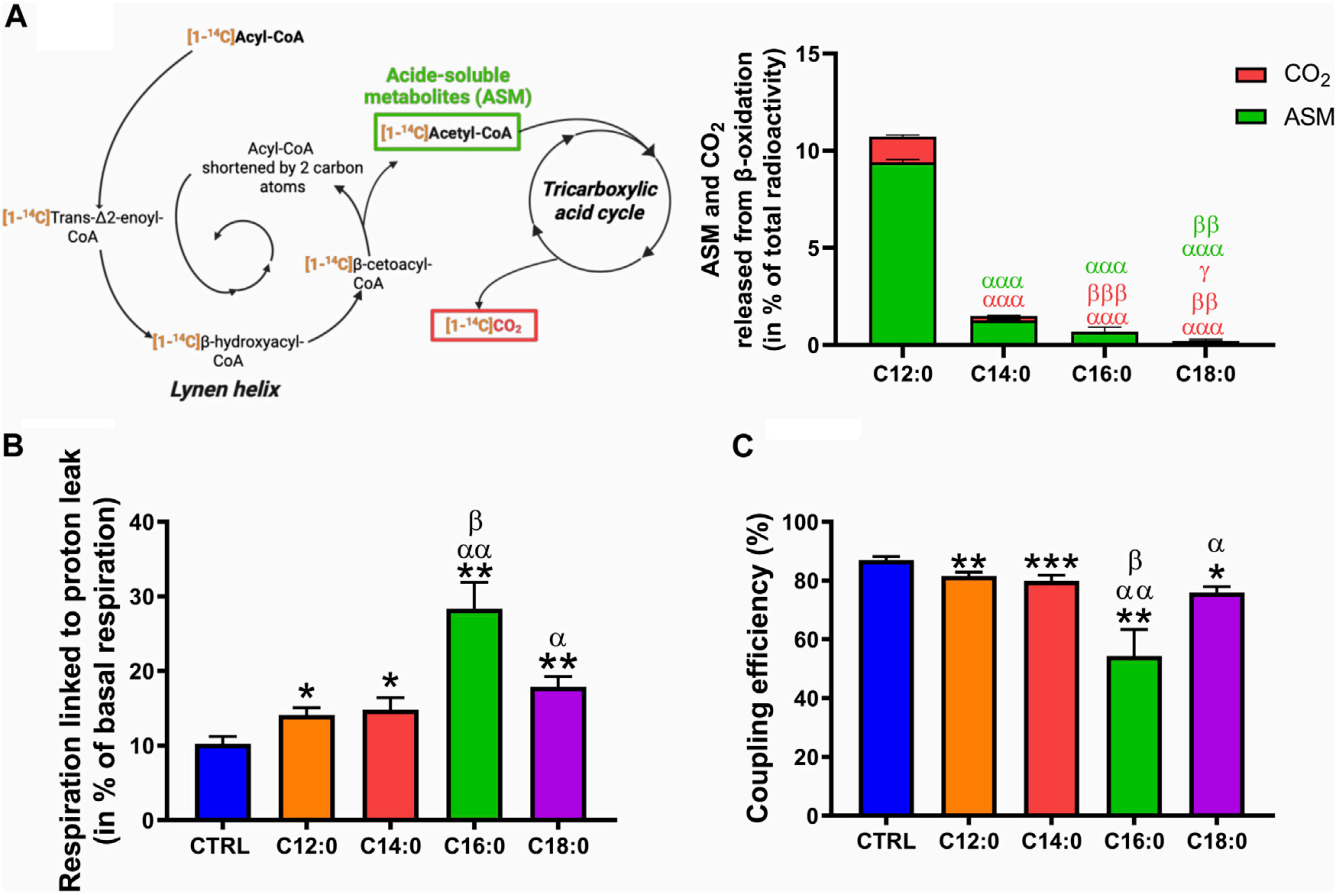
Fatty acid β-oxidation and OXPHOS uncoupling in response to fatty acid treatments. **(A)** ASMs and CO_2_ released from the β-oxidation of IPEC-J2 treated for 3 h with either [1–^14^C]C12:0, [1–^14^C]C14:0, [1–^14^C]C16:0, or [1–^14^C]C18:0 without prior chronic treatment. **(B)** Part of the basal respiration linked to proton leak and **(C)** OXPHOS coupling efficiency calculated from the Seahorse analysis of IPEC-J2 treated for 3 days with 250 µM of C12:0, C14:0, C16:0, or C18:0. N = 3–8 for CTRL, C12:0 and C16:0; N = 3–5 for C14:0; and N = 3 for C18:0 from independent experiments. Data are mean ± SEM. Significant differences are represented as *p < 0.05, **p < 0.01, and ***p < 0.001, vs. CTRL; ^α^P< 0.05, ^αα^P < 0.01, and ^ααα^P < 0.001 vs. C12:0; ^β^P < 0.05, ^ββ^P < 0.01, and ^βββ^P < 0.001 vs. C14:0; and ^γ^ *p* < 0.05 vs. C16:0.

Considering the increase in mitochondrial ROS production induced by C16:0 and C18:0, we hypothesized that elevated β-oxidation of these fatty acids may have enhanced ROS production, possibly through increased activity of the long-chain acyl-CoA dehydrogenase. To determine the ability of IPEC-J2 to β-oxidize fatty acids depending on their nature, the β-oxidation products (ASM and CO_2_) of each radiolabeled fatty acid incubated for 3 h on IPEC-J2 were measured without prior chronic treatment (Figure 7A). Interestingly, C12:0 was the most β-oxidized fatty acid since 9.4% ± 0.1% of the total [1–^14^C]C12:0 was converted into ASM and 1.3% ± 0.1% into CO_2_ after 3 h. C14:0 and C16:0 were significantly less β-oxidized, while C18:0 β-oxidation was barely detectable (ASM: 0.15% ± 0.15% and CO_2_: 0.06% ± 0.01%), indicating that C18:0 was not β-oxidized by the IPEC-J2 despite its entrance into the cells (Figure 7A). Increased proton leak in the mitochondrial inner membrane is also a mechanism that could lead to elevated ROS production. OXPHOS uncoupling and the part of the respiration lost in proton leak was, thus, calculated from OCR data and corresponds to the respiratory rate that persists despite the blocking of the ATP synthase by oligomycin (see OCR profiles in Figure 3A). In accordance with the observed reduced ATP production (Figure 4D), the respiration linked to proton leak was slightly increased by C12:0 and C14:0 (Figure 7B). Moreover, 18% to nearly 30% of the basal respiration of IPEC-J2 treated with C18:0 and C16:0, respectively, was lost by proton leak (Figure 7B). Hence, OXPHOS coupling efficiency was mildly diminished by C12:0 and C14:0, whereas it was severely impacted by C16:0 and C18:0 (Figure 7C).

## Discussion

Our objective was to evaluate if the nature of the dietary saturated fatty acids impacts IEC metabolism and barrier function in the *in vitro* model of intestinal epithelium IPEC-J2. We chose the four most consumed saturated fatty acids in human diets and compared their impact on IEC at similar concentrations, despite the fact that their consumption is not equal in diets (C16:0 > C18:0 > C14:0 > C12:0 in Western diets), to fully focus on their nature and not their quantity. By measuring TEER over time, we demonstrated that only C16:0 and C18:0 increased paracellular permeability contrary to the fatty acids with shorter-chain C12:0 and C14:0. In association with increased intestinal permeability, C16:0 and C18:0 induced severe mitochondrial alterations in enterocytes, marked by a diminution of ATP production due to high proton leak, OXPHOS uncoupling, mitochondrial network remodeling, and ROS generation, whereas C12:0 and C14:0 minimally altered these parameters and promoted mitochondrial fusion.

Several *in vitro* (Lindmark et al., 1998; Gulhane et al., 2016; Ghezzal et al., 2020; Gori et al., 2020; Weerakoon et al., 2021) as well as *in vivo* (Cani et al., 2009; Hamilton et al., 2015; Nascimento et al., 2021) studies have shown that high concentrations of fatty acid consumption/treatment increase paracellular permeability, indicating diminished epithelial barrier integrity in association with decreased expression of genes encoding tight junction proteins. Weerakoon et al. (2021) showed *in vitro* that C12:0 at 3 mM increased the translocation of chlorogenic acid compared to control on Caco-2 cells, suggesting enhanced intestinal permeability induced by C12:0. These results are strengthened by Lindmark *et al.* who demonstrated that C12:0 exhibited a dose-dependent effect on the passage of hydrophilic compounds across the Caco-2 monolayer (Lindmark et al., 1995; Lindmark et al., 1998). Nevertheless, C12:0 did not induce any redistribution of the tight junction proteins ZO-1 and occludin in Caco-2. It is noteworthy that the increase in intestinal permeability observed in those studies depends on the fatty acid nature and concentrations, as well as on the model used to measure intestinal permeability (Suzuki, 2013). We show here that 250 µM of C12:0 for 3 days did not modify IPEC-J2 TEER, suggesting maintaining in tight junction integrity in response to C12:0 as observed by Lindmark *et al.* Moreover, other studies mainly focused on a mix of fatty acids or on palmitic acid alone. We show here that C18:0, in addition to C16:0, caused a decrease in TEER, contrary to C12:0 and C14:0. However, we did not observe any change in the expression of genes encoding tight junction protein in IPEC-J2 treated with fatty acids. We rather propose that reduced TEER with C16:0 and C18:0 in our model of the intestinal epithelium is first caused by altered mitochondrial function and energy supply that support epithelial barrier integrity due to proton leak associated with elevated ROS production. It is noteworthy that C18:0 induced similar but less-pronounced alterations of the enterocyte bioenergetic and epithelial barrier than C16:0. This effect might be related to a lower absorption of C18:0 by IPEC-J2 compared to C16:0, as demonstrated by our uptake measurements. Others have already showed that C16:0 reduced respiratory rates associated with diminished OXPHOS-derived ATP production, mitochondrial membrane potential, and increased ROS generation in IEC *in vitro* (Sun et al., 2018; Li and Li, 2020; Yoo et al., 2021). Here, we demonstrated that these cell bioenergetic alterations and subsequent impact on barrier function are dependent on the fatty acid chain length, with long-chain fatty acids but not shorter ones being detrimental. Moreover, analysis of fatty acid composition of IPEC-J2 after treatment indicate that these enterocytes elongate C12:0 and C14:0 into C16:0 and C18:0 and even to C20:0, C22:0, and C24:0. However, the relative amount of these very-long-chain fatty acids is much lower compared to C16:0 and C18:0 measured in the cells and corresponds to less than 1% of the total fatty acids. We, thus, believe that C20:0, C22:0, and C24:0 are not likely to significantly contribute to the cellular alterations we observed compared to the higher proportions of C16:0 and C18:0. In addition to the elongation of C12:0 and C14:0 in C16:0 and C18:0, IPEC-J2 can also perform the desaturation of C16:0 into C16:1 (n-7) and that of C18:0 into C18:1 (n-9). Furthermore, we may wonder whether C16:0 and C18:0 obtained from the elongation of C12:0 and C14:0 could induce the moderate mitochondrial defects observed in IPEC-J2. However, since treatments with C12:0 and C14:0 did not severely impact mitochondrial function in IPEC-J2, we believe that the resultant C16:0 and C18:0 are likely to be stored in lipid droplets, as suggested by the significant Nile red staining after C12:0 or C14:0 treatment, and might consist in an adaptative mechanism to limit the lipotoxicity induced by high concentrations of free fatty acids.

Increased epithelial permeability might result from enhanced mitochondrial ROS generation that would potentiate tight junction damages. It has been indeed described that ROS alter epithelial barrier integrity through mechanisms linked with the redistribution of the tight junction proteins occludin (OCLN) and zonula occludens-1 (ZO-1) toward the intracellular compartment, the rearrangement of the actin cytoskeleton (Rao, 2008), or the phosphorylation of OCLN, causing its dissociation from ZO-1 (Gangwar et al., 2017). In contrast, treatment of IEC with antioxidants that target mitochondrial ROS *in vitro* (MitoTEMPO; mitoquinone) or *in vivo* (S3QELs) prevents the increase in epithelial permeability induced by the dinitrophenol-induced OXPHOS uncoupling (Wang et al., 2014; Lopes et al., 2018) or by high-fat diet consumption (Watson et al., 2021).

To explain the elevated ROS production induced by C16:0 and C18:0, we first hypothesized that enhanced mitochondrial β-oxidation of long-chain saturated fatty acids induced elevated superoxide anion production. Indeed, enzymes involved in the mitochondrial β-oxidation process are able to transfer electrons to oxygen to form ROS (Zhang et al., 2019). Hence, considering that the longer the fatty acid carbon chain, the greater the number of β-oxidation turns it requires to be oxidized, it would be reasonable to assume that C16:0 and C18:0 β-oxidation processes generate more ROS than fatty acids with shorter chains, such as C12:0 and C14:0. However, in accordance with previous works performed on rat hepatocytes (Rioux et al., 2003), we observed that C12:0 was rapidly oxidized by IPEC-J2, followed by C14:0, C16:0, and C18:0. Hence, the longer the carbon chain, the lower it was catabolized by enterocytes, thereby weakening our first hypothesis of enhanced ROS production due to greater C16:0 and C18:0 β-oxidation processes. Moreover, long-chain fatty acids have been shown to generate ROS in myotubes (Montgomery et al., 2013) or hepatocytes (Cardoso et al., 2013) through enhanced activity of the very-long-chain acyl-CoA dehydrogenase. The medium acyl-CoA dehydrogenases, involved in the β-oxidation of C12:0, do not generate ROS. Hence, high β-oxidation of C12:0 by medium acyl-CoA dehydrogenase is not likely to produce ROS (Cardoso et al., 2013). This enhanced C12:0 β-oxidation likely explains the elevated basal and maximal respirations of IPEC-J2 after 3 days of treatment.

In addition to ROS generation *via* β-oxidation, fatty acids also exert dual effects on mitochondrial respiration. It has been shown that long-chain fatty acids are natural OXPHOS uncouplers (Korshunov et al., 1998) that may block the activity of the respiratory chain and contribute to enhanced superoxide anion production. Indeed, whereas a mild uncoupling effect has been described as an adaptative mechanism to diminish ROS generation, higher uncoupling favors ROS production (Schönfeld and Wojtczak, 2007). Accordingly, we found that all the saturated fatty acids we tested on IPEC-J2 induced a significant increase in the respiratory rate linked to proton leak and, subsequently, a decrease in the OXPHOS coupling efficiency. This uncoupling effect was more pronounced after C16:0 or C18:0 treatments and may contribute to explain the low rates of ATP production. Hence, superoxide anion production induced by C16:0 and C18:0 might be caused by higher respiration linked to proton leak, compared to CTRL and C12:0/C14:0, and respiratory chain inhibition rather than through enhanced β-oxidation. Given that the OXPHOS uncoupler dinitrophenol increased intestinal permeability (JanssenDuijghuijsen et al., 2017) linked with oxidative stress and mitochondrial dysfunction (Nazli et al., 2004; Wang et al., 2014), a C16:0 or C18:0 treatment was likely to increase epithelial permeability through high OXPHOS uncoupling and associated ROS production.

Last, our mitochondrial network analysis indicated that all the fatty acid treatments induced remodeling of the mitochondrial network whether toward mitochondrial fusion in response to C12:0 and C14:0, as suggested by the greater average branch length of the network compared to CTRL cells or toward fission and fragmentation in response to C16:0 and C18:0, as suggested by the lower average branch length. This mitochondrial network fragmentation with C16:0 and C18:0 might be linked to increased mitochondrial ROS production since ROS modulate mitochondrial mass and dynamics and even promote mitochondrial fission (Ježek et al., 2020). Accordingly, a 24-h treatment with C16:0 of the IEC HCT116 disrupted the mitochondrial network, an effect that was prevented when cells were co-treated with the antioxidant N-acetyl-cysteine (Li and Li, 2020). In addition, in hepatocytes, enhanced mitochondrial fragmentation was associated with decreased fatty acid β-oxidation and mitochondrial respiration while ROS production was increased (Sebastián et al., 2012). Hence, in our model, increased mitochondrial ROS generation caused by C16:0 and C18:0 treatments may induce elevated mitochondrial fission in enterocytes and decreased ATP production.

In conclusion, although a treatment mechanism with C12:0 and C14:0 slightly uncoupled oxidative phosphorylation, provoking a mild decrease in ATP production, it did not alter tight junction integrity or the epithelial barrier. Contrastingly, a chronic treatment method with C16:0 or C18:0 provoked an important decrease in OXPHOS efficiency, as evidenced by proton leak associated with enhanced mitochondrial ROS production and mitochondrial network fragmentation, which ultimately depleted ATP production and increased epithelial permeability *in vitro*. We have to acknowledge that in our study, fatty acids were not provided through micelles but dissolved in DMSO and not in polarized cells, except for TEER measurement. Nonetheless, taken together, these data clearly advocate for further investigations into the role of dietary fatty acid composition on the mitochondrial function in enterocytes and their contribution to increased intestinal permeability *in vivo* and aggravation of different pathologies involving barrier function, such as inflammatory bowel disease or obesity. Moreover, it also opens ways to target mitochondrial function and ROS production in enterocyte with antioxidants that constitute a promising therapeutic target for treating intestinal barrier dysfunction.

## Data Availability

The raw data supporting the conclusion of this article will be made available by the authors, without undue reservation.

## Abbreviations

CS, citrate synthase; HFD, high-fat diet; IEC, intestinal epithelial cell; OXPHOS, oxidative phosphorylation; OCR, oxygen consumption rate; ROS, reactive oxygen species; TEER, transepithelial electrical resistance; TOMM20, translocase of outer mitochondrial membrane 20.
