# Supplementary material for: Saturated fatty acids differently affect mitochondrial function and the intestinal epithelial barrier depending on their chain length in the in vitro model of IPEC-J2 enterocytes
Source: Front Cell Dev Biol. 2024 Feb 1;12:1266842. doi: 10.3389/fcell.2024.1266842 (PMC10867211; doi:10.3389/fcell.2024.1266842)
Supplement: Supplementary file 1 [file Table1.DOCX]

Supplementary Material

|  | **Forward** | **Reverse** |
| --- | --- | --- |
| NADH:Ubiquinone Oxidoreductase Core Subunit 4 | TTATTGGTGCCGGAGGTACTG | CCCAGTTTATTCCAGGGTTCTG |
| (Nd4) |  |  |
| Succinate Dehydrogenase Subunit B | ACTGGATGGGCTGTACGAGT | GTCGATCATCCAGCGATAGG |
| (Sdhb) |  |  |
| Ubiquinol-Cytochrome C Reductase Core Protein 1 | TGCACCAGTGCCACAGAGA | GAGTGGTGCCATCCAGATGA |
| (Uqcrc1) |  |  |
| Cytochrome C Oxidase Subunit 4 | CCAAGTGGGACTACGACAAGAAC | CCTGCTCGTTTATTAGCACTGG |
| (Cox4) |  |  |
| ATP Synthase F1 Subunit Beta | GGGTACCATGCAGGAAAGAATC | GGCACATAGATGGCCTGTACAG |
| (Atp5b) |  |  |
| NAD(P)H Quinone Dehydrogenase 1 | CATGGCGGTCAGAAAAGCAC | ATGGCATACAGGTCCGACAC |
| (Nqo1) |  |  |
| Superoxide Dismutase 1 | AAGGCCGTGTGTGTGCTGAA | GATCACCTTCAGCCAGTCCTTT |
| (Sod1) |  |  |
| Glutathione Peroxidase 2 | AGAATGTGGCCTCGCTCTGA | GGCATTGCAGCTCGTTGAG |
| (Gpx2) |  |  |
| Catalase | TCCAGCCAGTGACCAGATGA | CCCGGTCAAAGTGAGCCATT |
| (Cat) |  |  |
| Occludin | ATGCTTTCTCAGCCAGCGTA | AAGGTTCCATAGCCTCGGTC |
| (Ocln) |  |  |
| Tight junction protein 1 | GGAGCATTGAAAGAAGCA | TGACAGGTAGGACAGACGA |
| (Tjp1) |  |  |
| Claudin 1 | AGATTTACTCCTACGCTGGTGAC | GCAAAGTGGTGTTCAGATTCAG |
| (Cldn1) |  |  |
| Claudin 2 | CATCCTCTGCTTTTCCTG | AACTCACTCTTGGCTTTGG |
| (Cldn2) |  |  |
| Claudin 7 | AGGCATCATTTTCATCGT | GACAAGAGCAAGAGAGCAG |
| (Cldn7) |  |  |
| TATA-Box Binding Protein | AACAGTTCAGTAGTTATGAGCCAGA | AGATGTTCTCAAACGCTTCG |
| (Tbp) |  |  |

**Supplementary table 1. Primers used for RT-qPCR**

|  | **CTRL** | **C12:0** | **C14:0** | **C16:0** | **C18:0** |
| --- | --- | --- | --- | --- | --- |
| *Nd4* | 1.02 ± 0.20 | 1.02 ± 0.34 | 0.83 ± 0.07 | 0.98 ± 0.28 | 0.81 ± 0.24 |
| *Sdhb* | 1.09 ± 0.49 | 1.05 ± 0.43 | 0.95 ± 0.45 | 1.06 ± 0.46 | 1.22 ± 0.55 |
| *Uqcrc1* | 1.39 ± 1.33 | 1.36 ± 0.69 | 1.09 ± 0.75 | 1.54 ± 1.21 | 1.98 ± 1.88 |
| *Cox4* | 1.42 ± 1.10 | 1.05 ± 1.06 | 0.74 ± 1.05 | 0.81 ± 0.79 | 0.47 ± 0.30 |
| *Atp5b* | 1.12 ± 0.59 | 0.94 ± 0.23 | 0.52 ± 0.21 | 0.75 ± 0.29 | 0.55 ± 0.12 |

**Supplementary table 2. Relative mRNA expression of genes encoding subunits of the electron transport chain.** Data are mean ± SEM.


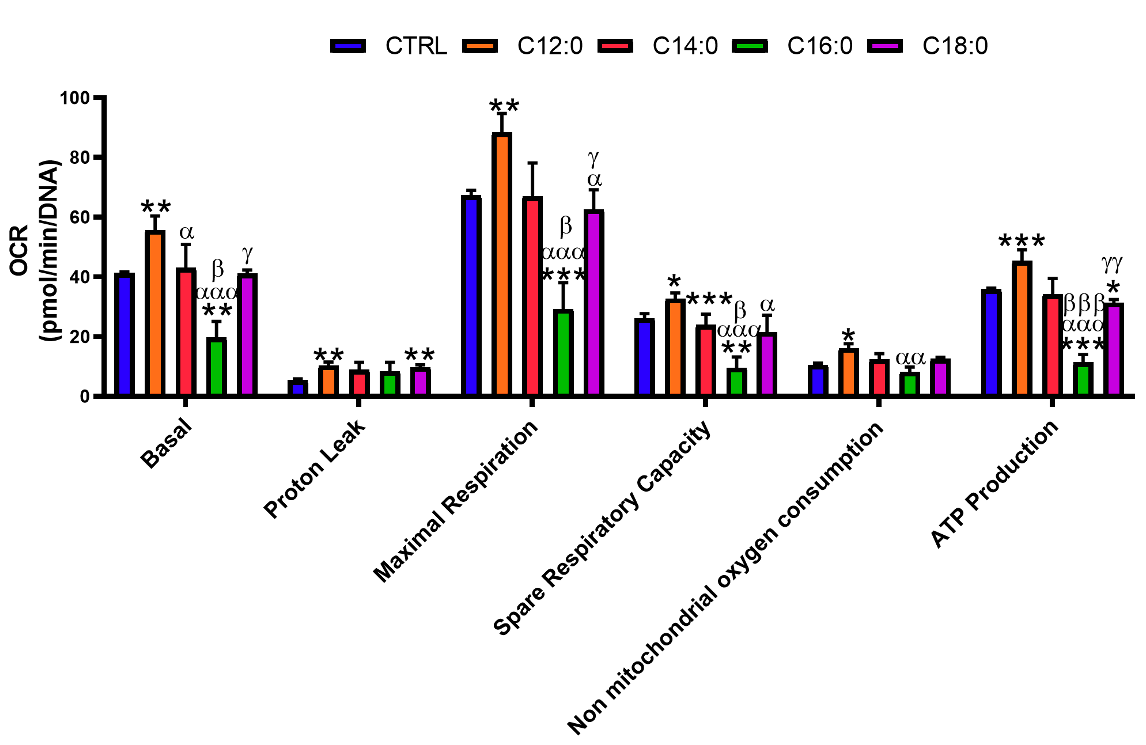


**Supplementary figure 1. Bioenergetic parameters in response to fatty acid treatment.** IPEC-J2 were treated for 3 days with 250 µM of C12:0. C14:0. C16:0 or C18:0 and the bioenergetic parameters were calculated from the OCR data. obtained from the Seahorse analyzer. and normalized by DNA content. Data are represented as mean ± SEM. Significant differences are represented as *P < 0.05, **P<0.01, ***P < 0.005 vs CTRL; ^α^P < 0.05, ^αα^P < 0.01 and ^ααα^P < 0.005 vs C12:0; ^β^P < 0.05 and ^βββ^P < 0.005 vs C14:0; ^γ^P < 0.05 and ^γγ^P < 0.01 vs C16:0.
